# (*E*)-*N*-[4-(Methyl­sulfon­yl)benzyl­idene]aniline

**DOI:** 10.1107/S1600536809050983

**Published:** 2009-12-04

**Authors:** Shao-Song Qian, Tao Liu

**Affiliations:** a School of Life Sciences, Shandong University of Technology, ZiBo 255049, People’s Republic of China

## Abstract

The mol­ecule of the title compound, C_14_H_13_NO_2_S, displays a *trans* configuration with respect to the C=N double bond. The dihedral angle between the two aromatic ring planes is 62.07 (18)°.

## Related literature

For a related structure, see: Qian & Cui (2009 ▶). For comparitive bond lengths, see: Allen *et al.* (1987 ▶).
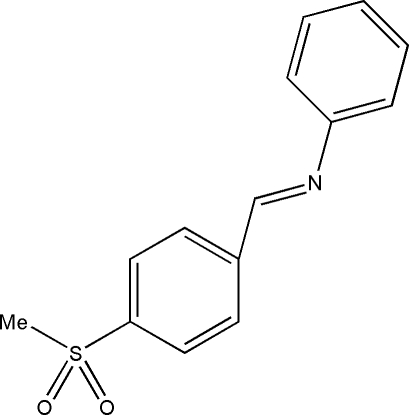


## Experimental

### 

#### Crystal data

C_14_H_13_NO_2_S
*M* *_r_* = 259.31
Monoclinic, 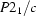

*a* = 8.2070 (16) Å
*b* = 5.7750 (12) Å
*c* = 26.945 (5) Å
β = 94.72 (3)°
*V* = 1272.7 (4) Å^3^
*Z* = 4
Mo *K*α radiation
μ = 0.25 mm^−1^
*T* = 293 K
0.30 × 0.20 × 0.10 mm

#### Data collection

Enraf–Nonius CAD-4 diffractometer
Absorption correction: multi-scan (*SADABS*; Sheldrick, 1996 ▶) *T*_min_ = 0.930, *T*_max_ = 0.976
2462 measured reflections
2292 independent reflections
1542 reflections with *I* > 2σ(*I*)
*R*_int_ = 0.053
3 standard reflections every 200 reflections intensity decay: 1%

#### Refinement

*R*[*F* ^2^ > 2σ(*F* ^2^)] = 0.052
*wR*(*F* ^2^) = 0.138
*S* = 1.03
2292 reflections
163 parameters
H-atom parameters constrained
Δρ_max_ = 0.29 e Å^−3^
Δρ_min_ = −0.34 e Å^−3^

### 

Data collection: *CAD-4 Software* (Enraf–Nonius, 1989 ▶); cell refinement: *CAD-4 Software*; data reduction: *XCAD4* (Harms & Wocadlo, 1995 ▶); program(s) used to solve structure: *SHELXS97* (Sheldrick, 2008 ▶); program(s) used to refine structure: *SHELXL97* (Sheldrick, 2008 ▶); molecular graphics: *SHELXTL* (Sheldrick, 2008 ▶); software used to prepare material for publication: *SHELXTL*.

## Supplementary Material

Crystal structure: contains datablocks global, I. DOI: 10.1107/S1600536809050983/rz2398sup1.cif

Structure factors: contains datablocks I. DOI: 10.1107/S1600536809050983/rz2398Isup2.hkl

Additional supplementary materials:  crystallographic information; 3D view; checkCIF report

## Figures and Tables

**Fig. 1.**
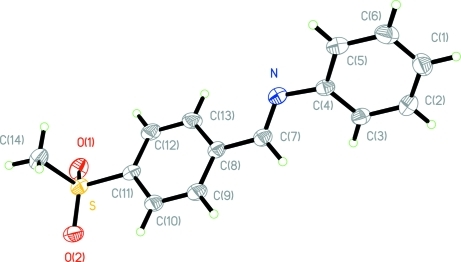
The structure of the title compound, showing the atom-labelling scheme. Displacement ellipsoids are drawn at the 35% probability level.

## supplementary crystallographic information

### Comment

Schiff base compounds have been of great of interest for many years. and act as important precursors for coordination chemistry related to catalysis and enzymatic reactions, magnetism and molecular archtectures. As an extension of work on the structural characterization of Schiff base compounds, the crystal structure of the title compound is reported here.

In the title compound (Fig. 1), all bond lengths are within normal ranges (Allen *et al.*, 1987) and comparable to the values observed in a closely related compound (Qian *et al.*, 2009). The molecule displays a trans-configuration with respect to the C=N double bond. The dihedral angle between two aromatic ring planes is 62.07 (18)°. The crystal packing is stabilized only by van der Waals interactions.

### Experimental

4-(Methylsulfonyl)benzaldehyde (0.184 g) and aniline (0.093 g) were dissolved in acetonitrile (20 ml). The mixture was stirred at room temperature for 10 min to give a clear yellow solution. After keeping the solution in air for 7 d, yellow block-shaped crystals suitable for X-ray analysis were formed at the bottom of the vessel on slow evaporation of the solvent.

### Refinement

All H atoms were placed in geometrical positions and constrained to ride on their parent atoms, with C—H = 0.93–0.96 Å, and with *U*_iso_(H) = 1.2 *U*_eq_(C) or 1.5 *U*_eq_(C) for methyl H atoms.

### Crystal data

**Table:**

C_14_H_13_NO_2_S	*F*(000) = 544
*M**_r_* = 259.31	*D*_x_ = 1.353 Mg m^−^^3^
Monoclinic, *P*2_1_/*c*	Mo *K*α radiation, λ = 0.71073 Å
Hall symbol: -P 2ybc	Cell parameters from 25 reflections
*a* = 8.2070 (16) Å	θ = 9–13°
*b* = 5.7750 (12) Å	µ = 0.25 mm^−^^1^
*c* = 26.945 (5) Å	*T* = 293 K
β = 94.72 (3)°	Block, yellow
*V* = 1272.7 (4) Å^3^	0.30 × 0.20 × 0.10 mm
*Z* = 4	

### Data collection

**Table:**

Enraf–Nonius CAD-4 diffractometer	1542 reflections with *I* > 2σ(*I*)
Radiation source: fine-focus sealed tube	*R*_int_ = 0.053
graphite	θ_max_ = 25.3°, θ_min_ = 1.5°
ω/2θ scans	*h* = 0→9
Absorption correction: multi-scan (*SADABS*; Sheldrick, 1996)	*k* = 0→6
*T*_min_ = 0.930, *T*_max_ = 0.976	*l* = −32→32
2462 measured reflections	3 standard reflections every 200 reflections
2292 independent reflections	intensity decay: 1%

### Refinement

**Table:**

Refinement on *F*^2^	Primary atom site location: structure-invariant direct methods
Least-squares matrix: full	Secondary atom site location: difference Fourier map
*R*[*F*^2^ > 2σ(*F*^2^)] = 0.052	Hydrogen site location: inferred from neighbouring sites
*wR*(*F*^2^) = 0.138	H-atom parameters constrained
*S* = 1.03	*w* = 1/[σ^2^(*F*_o_^2^) + (0.0501*P*)^2^ + 1.018*P*] where *P* = (*F*_o_^2^ + 2*F*_c_^2^)/3
2292 reflections	(Δ/σ)_max_ < 0.001
163 parameters	Δρ_max_ = 0.29 e Å^−^^3^
0 restraints	Δρ_min_ = −0.34 e Å^−^^3^

### Special details

**Table:**

Geometry. All esds (except the esd in the dihedral angle between two l.s. planes) are estimated using the full covariance matrix. The cell esds are taken into account individually in the estimation of esds in distances, angles and torsion angles; correlations between esds in cell parameters are only used when they are defined by crystal symmetry. An approximate (isotropic) treatment of cell esds is used for estimating esds involving l.s. planes.
Refinement. Refinement of F^2^ against ALL reflections. The weighted R-factor wR and goodness of fit S are based on F^2^, conventional R-factors R are based on F, with F set to zero for negative F^2^. The threshold expression of F^2^ > 2sigma(F^2^) is used only for calculating R-factors(gt) etc. and is not relevant to the choice of reflections for refinement. R-factors based on F^2^ are statistically about twice as large as those based on F, and R- factors based on ALL data will be even larger.

### Fractional atomic coordinates and isotropic or equivalent isotropic displacement parameters (Å^2^)

**Table:**

	*x*	*y*	*z*	*U*_iso_*/*U*_eq_	
S	0.80688 (10)	0.31400 (15)	0.45864 (3)	0.0473 (3)	
N	0.1198 (3)	−0.1536 (5)	0.34256 (10)	0.0474 (7)	
O1	0.9360 (3)	0.2295 (5)	0.43114 (9)	0.0609 (7)	
C1	−0.3341 (4)	−0.2970 (7)	0.26535 (15)	0.0635 (11)	
H1B	−0.4331	−0.3326	0.2477	0.076*	
O2	0.8001 (3)	0.5582 (4)	0.46914 (11)	0.0680 (8)	
C2	−0.2463 (5)	−0.1064 (7)	0.25292 (15)	0.0592 (10)	
H2B	−0.2866	−0.0125	0.2267	0.071*	
C3	−0.0992 (4)	−0.0518 (6)	0.27874 (13)	0.0488 (9)	
H3A	−0.0420	0.0793	0.2701	0.059*	
C4	−0.0363 (4)	−0.1919 (6)	0.31746 (12)	0.0426 (8)	
C5	−0.1259 (4)	−0.3852 (6)	0.32996 (14)	0.0537 (9)	
H5A	−0.0857	−0.4813	0.3558	0.064*	
C6	−0.2737 (5)	−0.4344 (7)	0.30415 (16)	0.0632 (11)	
H6A	−0.3334	−0.5624	0.3131	0.076*	
C7	0.1609 (4)	0.0524 (6)	0.35336 (12)	0.0467 (8)	
H7A	0.0855	0.1699	0.3458	0.056*	
C8	0.3215 (4)	0.1149 (6)	0.37721 (12)	0.0433 (8)	
C9	0.3412 (4)	0.3236 (6)	0.40197 (15)	0.0586 (10)	
H9A	0.2534	0.4255	0.4017	0.070*	
C10	0.4877 (4)	0.3840 (6)	0.42707 (14)	0.0564 (10)	
H10A	0.4982	0.5241	0.4441	0.068*	
C11	0.6192 (4)	0.2351 (6)	0.42680 (12)	0.0435 (8)	
C12	0.6034 (4)	0.0265 (6)	0.40118 (14)	0.0549 (10)	
H12A	0.6925	−0.0726	0.4004	0.066*	
C13	0.4555 (4)	−0.0325 (6)	0.37695 (14)	0.0537 (9)	
H13A	0.4447	−0.1729	0.3601	0.064*	
C14	0.8121 (4)	0.1617 (7)	0.51489 (13)	0.0603 (10)	
H14A	0.9119	0.1963	0.5346	0.090*	
H14B	0.8065	−0.0015	0.5082	0.090*	
H14C	0.7206	0.2067	0.5327	0.090*	

### Atomic displacement parameters (Å^2^)

**Table:**

	*U*^11^	*U*^22^	*U*^33^	*U*^12^	*U*^13^	*U*^23^
S	0.0448 (5)	0.0432 (5)	0.0537 (6)	0.0085 (4)	0.0032 (4)	0.0064 (4)
N	0.0488 (16)	0.0410 (17)	0.0523 (17)	0.0068 (14)	0.0040 (13)	0.0032 (14)
O1	0.0459 (13)	0.0773 (19)	0.0610 (16)	0.0114 (13)	0.0130 (11)	0.0111 (14)
C1	0.053 (2)	0.066 (3)	0.071 (3)	−0.004 (2)	−0.0011 (19)	−0.007 (2)
O2	0.0654 (16)	0.0407 (14)	0.094 (2)	0.0054 (13)	−0.0148 (15)	−0.0008 (14)
C2	0.058 (2)	0.060 (3)	0.059 (2)	0.010 (2)	−0.0002 (19)	0.007 (2)
C3	0.053 (2)	0.0402 (19)	0.054 (2)	0.0019 (17)	0.0059 (17)	0.0059 (17)
C4	0.0486 (19)	0.0355 (18)	0.0449 (19)	0.0070 (16)	0.0099 (15)	−0.0033 (16)
C5	0.062 (2)	0.040 (2)	0.060 (2)	0.0055 (18)	0.0112 (19)	0.0028 (17)
C6	0.064 (2)	0.047 (2)	0.081 (3)	−0.008 (2)	0.015 (2)	−0.001 (2)
C7	0.050 (2)	0.043 (2)	0.047 (2)	0.0093 (16)	0.0067 (16)	−0.0008 (17)
C8	0.0459 (19)	0.0406 (19)	0.0437 (19)	0.0078 (15)	0.0044 (15)	0.0014 (15)
C9	0.050 (2)	0.045 (2)	0.079 (3)	0.0195 (18)	−0.0019 (19)	−0.008 (2)
C10	0.052 (2)	0.042 (2)	0.074 (3)	0.0153 (17)	−0.0066 (19)	−0.0130 (19)
C11	0.0469 (18)	0.0373 (19)	0.047 (2)	0.0096 (15)	0.0088 (15)	0.0063 (15)
C12	0.048 (2)	0.044 (2)	0.073 (3)	0.0183 (17)	0.0069 (19)	−0.0034 (19)
C13	0.054 (2)	0.039 (2)	0.068 (2)	0.0087 (17)	0.0065 (18)	−0.0107 (18)
C14	0.061 (2)	0.066 (3)	0.054 (2)	0.018 (2)	0.0050 (18)	0.008 (2)

### Geometric parameters (Å, °)

**Table:**

S—O1	1.428 (2)	C6—H6A	0.9300
S—O2	1.440 (3)	C7—C8	1.463 (5)
S—C14	1.750 (4)	C7—H7A	0.9300
S—C11	1.760 (4)	C8—C9	1.381 (5)
N—C7	1.264 (4)	C8—C13	1.391 (4)
N—C4	1.416 (4)	C9—C10	1.375 (5)
C1—C2	1.372 (5)	C9—H9A	0.9300
C1—C6	1.372 (5)	C10—C11	1.380 (4)
C1—H1B	0.9300	C10—H10A	0.9300
C2—C3	1.379 (5)	C11—C12	1.389 (5)
C2—H2B	0.9300	C12—C13	1.373 (5)
C3—C4	1.386 (4)	C12—H12A	0.9300
C3—H3A	0.9300	C13—H13A	0.9300
C4—C5	1.393 (5)	C14—H14A	0.9600
C5—C6	1.377 (5)	C14—H14B	0.9600
C5—H5A	0.9300	C14—H14C	0.9600
			
O1—S—O2	118.61 (17)	N—C7—H7A	118.4
O1—S—C14	108.15 (16)	C8—C7—H7A	118.4
O2—S—C14	108.70 (19)	C9—C8—C13	118.4 (3)
O1—S—C11	108.39 (15)	C9—C8—C7	119.6 (3)
O2—S—C11	107.63 (15)	C13—C8—C7	122.1 (3)
C14—S—C11	104.47 (17)	C10—C9—C8	121.5 (3)
C7—N—C4	118.1 (3)	C10—C9—H9A	119.3
C2—C1—C6	119.1 (4)	C8—C9—H9A	119.3
C2—C1—H1B	120.5	C9—C10—C11	119.4 (3)
C6—C1—H1B	120.5	C9—C10—H10A	120.3
C1—C2—C3	121.0 (4)	C11—C10—H10A	120.3
C1—C2—H2B	119.5	C10—C11—C12	120.1 (3)
C3—C2—H2B	119.5	C10—C11—S	119.3 (3)
C2—C3—C4	120.1 (3)	C12—C11—S	120.6 (2)
C2—C3—H3A	119.9	C13—C12—C11	119.6 (3)
C4—C3—H3A	119.9	C13—C12—H12A	120.2
C3—C4—C5	118.6 (3)	C11—C12—H12A	120.2
C3—C4—N	122.3 (3)	C12—C13—C8	121.0 (3)
C5—C4—N	119.0 (3)	C12—C13—H13A	119.5
C6—C5—C4	120.2 (4)	C8—C13—H13A	119.5
C6—C5—H5A	119.9	S—C14—H14A	109.5
C4—C5—H5A	119.9	S—C14—H14B	109.5
C1—C6—C5	120.9 (4)	H14A—C14—H14B	109.5
C1—C6—H6A	119.6	S—C14—H14C	109.5
C5—C6—H6A	119.6	H14A—C14—H14C	109.5
N—C7—C8	123.2 (3)	H14B—C14—H14C	109.5
			
C6—C1—C2—C3	0.1 (6)	C8—C9—C10—C11	1.2 (6)
C1—C2—C3—C4	1.0 (6)	C9—C10—C11—C12	0.3 (6)
C2—C3—C4—C5	−1.0 (5)	C9—C10—C11—S	179.6 (3)
C2—C3—C4—N	175.0 (3)	O1—S—C11—C10	−144.6 (3)
C7—N—C4—C3	42.2 (5)	O2—S—C11—C10	−15.2 (3)
C7—N—C4—C5	−141.8 (3)	C14—S—C11—C10	100.2 (3)
C3—C4—C5—C6	0.0 (5)	O1—S—C11—C12	34.7 (3)
N—C4—C5—C6	−176.1 (3)	O2—S—C11—C12	164.1 (3)
C2—C1—C6—C5	−1.0 (6)	C14—S—C11—C12	−80.5 (3)
C4—C5—C6—C1	1.0 (6)	C10—C11—C12—C13	−1.3 (5)
C4—N—C7—C8	−177.2 (3)	S—C11—C12—C13	179.5 (3)
N—C7—C8—C9	−159.4 (4)	C11—C12—C13—C8	0.7 (6)
N—C7—C8—C13	18.9 (5)	C9—C8—C13—C12	0.7 (6)
C13—C8—C9—C10	−1.7 (6)	C7—C8—C13—C12	−177.6 (3)
C7—C8—C9—C10	176.6 (4)		
